# Therapeutic Patient Education for Fibromyalgia during Spa Therapy: The FiETT Randomized Controlled Trial

**DOI:** 10.3390/ijerph19084613

**Published:** 2022-04-11

**Authors:** Philippe Ducamp, Patrick Sichère, Hermine Gayum, Karine Dubourg, Christian-François Roques, Valérie Journot

**Affiliations:** 1 Physical & Rehabilitation Medicine Department, Orthez Hospital, 64300 Orthez, France; 2 Rheumatology Department, Delafontaine Hospital, 93200 Saint-Denis, France; patrick.sichere@gmail.com; 3 ISPED School of Public Health, Bordeaux University, 33000 Bordeaux, France; hermine.waffo-teguo@u-bordeaux.fr; 4 Balneology Institute, Bordeaux University, 40100 Dax, France; karine.dubourg@u-bordeaux.fr; 5 Physical & Rehabilitation Medicine Department, Toulouse III Paul Sabatier University, 31062 Toulouse, France; cf.roques@gmail.com; 6 Bordeaux Population Health, INSERM, 33000 Bordeaux, France; valerie.journot@inserm.fr

**Keywords:** fibromyalgia, spa therapy, therapeutic patient education, fibromyalgia impact questionnaire, randomized trial

## Abstract

Spa therapy is known to improve quality of life and diminish pain. We assessed the efficacy (Fibromyalgia Impact Questionnaire-FIQ) and safety at 6 months of a fibromyalgia-specific therapeutic patient education (TPE) program added to fibromyalgia-specific standardized spa therapy (SST), compared to SST alone, in a controlled randomized trial. We enrolled 157 patients, mostly women, attending spa centers in Southwest France in 2015–2016, and randomized them to SST + TPE (79) or SST (78). The intention-to-treat with “missing as failure” analysis showed a tendency toward a higher, though non-significant, benefit with TPE than without for FIQ (−9 vs. −3; *p* = 0.053) or pain intensity (−0.9 vs. −1.1; *p* = 0.58). In addition, pain relief (+3.2 vs. +4.3; *p* = 0.03) and fatigue (−1.6 vs. −3.7; *p* = 0.02) were significantly improved, and 87% patients in the SST + TPE arm still regularly practiced the physical exercises taught to them at 6 months. We suspect significant and lasting improvement from spa therapy, as well as our already well-informed and well-managed participants, to have prevented the demonstration of a significant benefit of TPE on FIQ.

## 1. Introduction

Fibromyalgia impacts domestic, leisure, and professional abilities [1]. Severity is related to the disease’s psycho-social impact [2,3,4,5]; genetic polymorphism may modulate pain sensitivity, mood, perception and response to treatment [6,7,8,9,10,11,12,13]; depression [14] and physical or psychological trauma contribute to disease onset or continuation. Fibromyalgia, with a prevalence of 1.6% in France [15], occurs between 35 and 55 years of age and 70 to 90% of those affected are women [4,16]. Diagnostic criteria evolved between 1990 and 2010 [5,17,18], then after 2016 [19], and the non-specificity of etiology and symptoms makes the diagnosis unrecognized by some doctors. The European Alliance of Associations for Rheumatology (EULAR) recommends non-pharmacological interventions (exercise, physiotherapy, balneotherapy, acupuncture, mind-body techniques, etc.) [20,21] as first-line because of insufficient data, a limited response, and poor tolerance to drugs used alone or in combination (analgesics, antiepileptics, antidepressants, etc.) [22].

Spa therapy (ST), widely prescribed in Europe, Japan, South America, and North Africa, is recommended for fibromyalgia for its analgesic, relaxing effects and supervised exercise. Randomized controlled trials suggest a benefit of ST on pain [8,23,24,25,26,27,28,29,30,31,32,33,34,35,36], quality of life measured by the Fibromyalgia Impact Questionnaire (FIQ) [24,26,27,29,30,35,37,38] or assessed with generic scales (BDI, SF-36, VAS, PSQI, MPQ, CIS, GTPS) [24,35,38], mood [8,24,25,31,35], sleep [8,34,39], fatigue [8,25,34], gastric dyspepsia [25,34], irritable bowel syndrome [8,25], and dyspnea of respiratory origin [31]. Reviews and meta-analyses confirmed these observations but emphasized the low power of some trials [10,21,23,28,32,40,41,42,43,44,45,46,47]. Nevertheless, EULAR stated that: “hot-water baths, with or without exercise, are effective in fibromyalgia because they improve pain and function” [20]. Using mineral water was better than using tap water [24,29,31] even if the care provided was not daily [26]. The effect size of ST (modified Cohen’s d) was 1.408 on pain and 2.085 on function [16], and a weighted differential effect size was calculated at 0.736 for the reduction of pain intensity [48].

In chronic diseases, therapeutic patient education (TPE) aims to change individual health-related behavior. According to the World Health Organization (WHO), TPE is “continuous, integrated, patient-centered, including awareness-raising, information, self-care learning, psychosocial support activities regarding the disease, prescribed treatment, care, hospitalization, behavior related to health and illness” [49]. In France, TPE programs have specifications for implementation and assessment and are controlled by public health agencies. Fibromyalgia TPE programs have been implemented in hospital or university settings [44,50,51,52,53,54], balneotherapy facilities [55,56], via the internet [57], or using written documents at home [51]. Some of these studies are uncontrolled [53,54,57], but randomized controlled trials show an impact on the symptoms [44,50,51]. In France, ST has a duration imposed by the Ministry of Health of 3 (or 2) weeks and is dedicated to care, rehabilitation, and education. The addition of ST to an educational program, compared to an educational program alone, significantly lessened the symptoms of fibromyalgia [55,56]. Fibr’Eaux®, a fibromyalgia-specific TPE program, running since 2014 in the Dax spa facilities, aims to improve patient quality of life through an accurate and adapted comprehension of their situation, optimal use of drugs, and better level of physical activity. We hypothesized that Fibr’Eaux® delivered during a 3-week ST would reduce, compared to ST alone, the burden of fibromyalgia at 6 months after ST assessed through the Fibromyalgia Impact Questionaire.

## 2. Methods

### 2.1. Design

FiETT (Fibromyalgie et Education Thérapeutique en cure Thermale) was a multicenter open-label randomized trial comparing the impact on fibromyalgia symptoms of the Fibr’Eaux^®^ TPE program added to standardized fibromyalgia-specific spa therapy (SST + TPE) vs. standardized fibromyalgia-specific spa therapy only (SST) at 6 months after spa therapy.

### 2.2. Recruitment

The FiETT trial was realized in 12 thermal spa establishments (See Acknowledgments section) in Southwest France. Patients were recruited through doctors in the Dax/St Paul les Dax resort, the spa establishments, or via several websites, particularly patient association sites. The Dax Information Center set up a hotline giving information about the trial, participating facilities, and investigators participating in the trial. Fifteen 3-week sessions were organized between March 2015 and November 2016.

### 2.3. Participants

Inclusion criteria were: age between 18 and 65; fibromyalgia diagnosed at least 6 months before screening (baseline) and confirmed by clinical criteria [18] and the Fibromyalgia Rapid Screening Test (FiRST) [58] (score 5 or 6 at baseline) checked by the investigator; internet access from end of spa therapy to end of remote follow-up (day 19 to month 12); French health insurance; and voluntarily signing an informed consent form. Non-inclusion criteria included: infectious disease with ongoing treatment (except for minor seasonal upper respiratory tract infections); active cancer; concomitant polymyalgia rheumatica; stage III venous insufficiency with a history of deep venous thrombosis or pulmonary embolism; contraindication to warm hydrotherapy (≥36 °C); total inability to practice the physical activities planned in the program; ongoing pregnancy; participation in a fibromyalgia-specific TPE program during year before baseline; and expected difficulties to fill out self-administered questionnaires.

### 2.4. Interventions

At baseline, all patients were prescribed a practice-designed, validated, and consensual spa therapy program consisting of 72 treatments over 18 consecutive days except Sunday/3 weeks except Sunday, adapted to their tolerance level, including: individual baths in mineral water with underwater spray (36 °C, 10–20 min), mineral mud applications (40–42 °C, up to 15 min), massages by a registered physiotherapist (36 °C, up to 10 min), and collective exercises in a mineral water pool supervised by a registered physiotherapist (32–34 °C, 20 min). The Dax resort’s natural waters are hyper-thermal (gushing at 54 to 62 °C) and moderately mineralized (1 g/L) with calcium sulfate (410 mg/L), sodium chloride (226 mg/L), magnesium sulfate (116 mg/L), and oligo metallic chlorides. Dax peloids consist of stilt from the Adour River matured with mineral water and blue-green algae. The intervention is delivered by a shared team and the staff of the spa are all qualified.

Patients received hydro-mineral treatments in the morning. In the afternoon, those allocated to the SST + TPE group attended 1.5–2 h collective educational workshops (8 to 12 attendees) in the Dax Balneology Institute (Bordeaux University), taking advantage of the human and material resources offered by a spa resort. The program includes: (i) an initial joint educational and physical assessment by the spa doctor, TPE nurse, and a trained physical education coach, so as to adapt the program to the patient’s limitations; (ii) collective educational workshops with supervised physical activities; (iii) an educational and physical assessment at the end of the intervention; and (iiii) an “action plan” for the patient to be implemented back home with potential adaptation as needed. (See Supplemental File for details—Table S1). Four workshops were mandatory (Understanding the Disease, Managing Pain and Stress, Moving Well, and Sleeping Well) and two were optional (Using Drugs Well and Living with Disease). They also practiced three collective 1 h indoor sessions of stretching and relaxation exercises adapted to patients with fibromyalgia, and three collective outdoor sessions of adapted aerobic activity (walking 500 to 1000 m, punctuated by eight exercise stations and three rest stations). Indoor and outdoor sessions were supervised by a trained adapted-physical-activity coach. To prevent contamination between patients allocated to the different strategies and to minimize disappointment bias the program was proposed free of charge to all patients allocated to the SST group to take place in the year following month 12. Missing patient self-reported data in both groups were minimized by giving a EUR 100 (USD 110) incentive to all patients completing questionnaires at months 3, 6, and 12. In addition, we thoroughly described painful episodes of fibromyalgia and adverse events.

### 2.5. Follow-Up

The enrollment (baseline) visit took place before spa therapy began. Investigators screened potential patients for eligibility, provided them with information about the trial, ensured informed consent, and randomized them to either SST + TPE or SST alone. The course of daily spa therapy started on a Monday and continued for 3 weeks (except Sundays). Investigators performed fibromyalgia and spa therapy checkups at days 9 and 18. During the spa therapy course, the spa nurse continuously recorded any adverse event of interest and any deviation from scheduled care. All data relative to spa therapy were entered in an electronic case report form (eCRF) by investigators, the spa nurse, TPE nurse, and coach. At day 18, every day patients filled in a paper logbook containing self-reported questionnaires on domains recommended by the outcome measures in rheumatology (OMERACT, Home made eCRF created by the Medical Informatics Research and Development Center of the University of Bordeaux, *Bordeaux*, France) [59,60]: pain intensity and relief, kinesiophobia, pain catastrophizing, functional limitations, sleepiness and fatigue, anxiety and depression, compliance with prescribed spa care, and compliance with the TPE program for patients in the SST + TPE group. Following the spa therapy course, patients answered the same self-reported questionnaires as in the eCRF, online at months 3, 6, and 12. The self-monitoring logbook with the self-reported questionnaire was preferred to interrogations that may induce answers. Patients received automatic emails and phone calls from spa nurses to remind them to complete the self-administered questionnaires. Spa nurses collected adverse events and pregnancies by phone. Events of interest were painful episodes, any serious adverse event including death, and eventual study withdrawal. All data and procedures during the spa therapy course were monitored on site by a trial CRA. All data collected during follow-up were monitored for response, but not for completeness or exactness since no corrective action could be undertaken.

### 2.6. Outcomes

The primary outcome was the mean change between baseline and month 6 on the FIQ score, ranging from 0 (no impact) to 100 (highest impact) [61,62]. The main secondary outcome was the rate of response, defined as a relative change in FIQ between baseline and month 6 strictly below 14%. Other secondary outcomes were domains recommended by OMERACT [60] as scores measured using the self-administered questionnaires at 3, 6, and 12 months after spa therapy: changes from baseline in FIQ score [61,62], pain intensity and relief assessed via Visual Analog Scales (VAS) [63,64], kinesiophobia [65,66,67], pain catastrophizing [68], functional limitations [69], fatigue [70], sleepiness [39,71], anxiety, and depression [72]. We also measured the change from baseline in non-pharmacological or pharmacological treatments recommended or discouraged by EULAR [73]. Serious adverse reactions and painful episodes were validated by an independent adjudication committee of two rheumatologists and one specialist in pharmacoepidemiology, masked to the trial group.

### 2.7. Sample Size

This was a superiority trial. The minimal clinically important change in FIQ score between interventions estimated in three previous randomized trials was 9 points, i.e., 14% [74].Consequently we considered a difference of 10 points between strategies to be relevant. Assuming a normal distribution of FIQ change between baseline and month 6 and a common standard deviation of 20 points, a (1:1) sample size of 64 patients per strategy would have 80% power to detect a difference of 10 points using a Student test with a 0.05 two-sided significance level (nQuery Advisor^®,^ nQuery Advisor v7.0, Statistical Solutions, Cork, Ireland). We anticipated a maximum of 15% missing values concerning the change in FIQ score, so the sample size was set at 152 patients.

### 2.8. Randomization

Due to a possible difference in the influence of the staff at the various spa facilities on patient compliance and outcomes, randomization was stratified by thermal spa facility (balance rate 1:1, four-patient blocks). The randomization list was generated by the statistician with SAS 9.4-proc-plan^®^ and implemented in the eCRF. After obtaining informed consent, the screening examination, and inclusion, investigators entered the patient’s data, performed randomization, and informed the patients of their allocated strategy. Spa nurses, and the TPE nurse and educators for SST + TPE strategy, were informed by a computer-generated email.

### 2.9. Statistical Methods

Primary analysis was performed on intention-to-treat, with missing values imputed by a failure value defined by protocol as the first decile in the arm of the corresponding patient. We first checked the absence of a site effect through a mixed linear model on FIQ change at month 6. Then we compared strategies with a Student test. We performed a twofold worst-case sensitivity analysis, with missing values in an arm imputed alternatively by first (failure) or nineth (success) decile in the same arm [European Medicines Agency. Guidelines on missing data in confirmatory clinical trials]. https://www.ema.europa.eu/en/documents/scientific-guideline/guideline-missing-data-confirmatory-clinical-trials_en.pdf (access date 4 January 2022).

We conducted secondary analyses on observed data: on-treatment analysis, and mixed linear model on FIQ over time with between-strategies comparison of estimated slopes during spa therapy and remote follow-up. In view of the limited effectiveness of ETP in bringing about an improvement in the patient’s condition, it seemed interesting to us, in post hoc analysis, to try to identify sub-populations that respond better to the intervention, in order to define for whom this intervention may be most beneficial. We searched for the determinants of FIQ response among the patients’ baseline demographic, social, and clinical characteristics, non-FIQ scores and limited range of joint motion, using a logistic regression model with univariable (significance level 20%) and multivariable (backward selection, significance level 5%) analyses. We compared strategies regarding secondary outcomes with Student or Wilcoxon, chi-square or exact tests, depending on endpoint distribution. To account for correlation between non-FIQ scores, we used a multivariate analysis of variance to test the global effect of the strategy on change in scores from baseline. We described the nature and causality of serious adverse events and reactions. We compared strategies on the dynamics of painful episodes using a Kaplan–Meier curve and a log-rank test. We described episodes of symptoms and searched for their determinants by the above selection methods. We used mixed linear models to confirm the overall efficacy of spa therapy during the spa therapy course and afterward using EULAR criteria: pain intensity, fatigue, sleepiness, and disability (assessed by FIQ) [73]. We also described compliance to spa therapy treatments and to the TPE program during the spa therapy course and follow-up.

### 2.10. Ethics and Authorizations

FiETT was sponsored and funded (grant n° C2013/02), by the French Society for Thermal Spa Research (Association Française pour la Recherche Thermale—AFRETh), an independent non-profit organization. It was approved by the local ethics committee (Comité de Protection des Personnes Sud-Ouest et Outre-Mer, decision n° 2014-A00464-43), the competent French authority (Agence Nationale de Sécurité du Médicament et des produits de santé, ref. 140815B-32), and the French data protection authority (Commission Nationale de l’Informatique et des Libertés, decision DR-2014-317). It was registered on ClinicalTrials.gov (n° NCT02406313) before the trial started.

## 3. Results

### 3.1. Enrolment and Follow-Up

Among 164 patients screened between March 2015 and November 2016, 157 fulfilled all eligibility criteria and were randomized to receive SST (*n* = 78) or SST + TPE (*n* = 79) strategies (See in Supplemental File Figure S1). Eight patients withdrew consent: five (6%) in SST vs. three (4%) in SST + TPE groups, five during spa therapy and three during follow-up. Three more patients were lost to follow-up in each strategy. These events did not differ between strategies (*p* = 0.59). All patients started the allocated strategy at baseline but 29 (18%) discontinued it: four (5%) in SST vs. 25 (32%) in SST + TPE, *p* < 10^−4^. The reasons for discontinuation were painful episodes in five (3 vs. 2), adverse events for three (0 vs. 3), and patient’s choice for 21 (1 vs. 20) for SST and SST + TPE strategies, respectively.

### 3.2. Patients’ Characteristics

Patients did not differ in baseline characteristics between strategies (Table 1). They were mainly women in their fifties, 50% were in employment and 71% had moderate to high income. Their average body mass index was 26 kg/m^2^ (Standard Error (SE) 2) but 22% suffered from severe or morbid obesity. About one-third had already undergone musculoskeletal surgery (19%) or experienced other musculoskeletal disorders (30%). Most had a maximum FIRST score (85%). The average FIQ score was 60 points/100 (SE 1), with an average pain intensity of 7 points/10 (SE 0). During the last 3 months before baseline, some patients had no non-pharmacological treatment at all (21% vs. 32% for SST and SST + TPE strategies, respectively); others had physical therapy (65% vs. 57%, resp.), osteopathy (13% vs. 4%, resp.), acupuncture (19% vs. 14%, resp.), relaxation (15% vs. 9%, resp.), hypnosis (13% vs. 10%, resp.), or other non-pharmacological intervention (38% vs. 25%, resp.). Some patients took no drugs at all (17%), took the drugs recommended, and avoided the drugs strongly discouraged by the European Alliance of Associations for Rheumatology (EULAR) (41%), or disrespected EULAR recommendations (41%: 8% took discouraged drugs and non-recommended drugs; 10% took both; 24% took none of them) [73].

### 3.3. Fibromyalgia Impact Questionnaire

**Figure 1:**
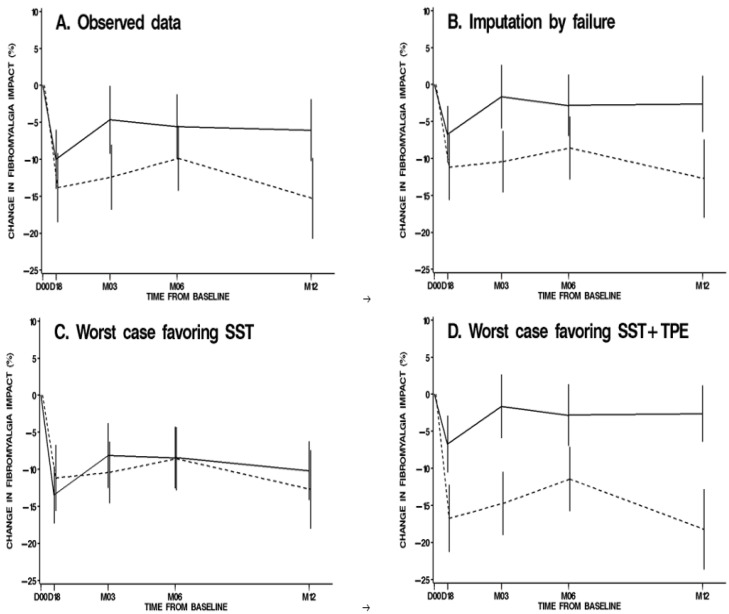
Mean changes from baseline (95% confidence interval) in Fibromyalgia Impact Questionnaire, on intention to treat analysis according to missing values imputation method—FiETT trial, AFRETh. SST (solid line): standard spa therapy; SST + TPE (dashed line): standard spa therapy + therapeutic patient education. Missing values were imputed by decile values observed in the same strategy (1st = success; 9th = failure), as protocoled.

We observed 15 items of missing data at month 6 (10%), less than the expected 15%. The amount of missing data was higher with the SST than in SST + TPE strategy (13% vs. 6%, *p* = 0.17) (See Supplemental File, Table S2). In the intention-to-treat analysis of change in FIQ month 6 with missing values replaced by “failure” (78 vs. 79 patients included), there was no site effect (*p* = 0.18) and a non-significant difference between strategies (−3 points (SE 2) for SST vs. −9 (SE 2) for SST + TPE, *p* = 0.053) (Figure 1). With 157 patients and an observed difference of −6 points, the power of the Student test was only 48% (it would have been 91% if the expected difference of −10 points had been reached). The worst-case sensitivity analysis was inconclusive: −8 (SE 2) vs. −9 (SE 2), *p* = 0.96 when favoring SST alone; and −3 (SE 2) vs. −11 (SE 2), *p* = 0.004 when favoring the SST + TPE strategy. For the on-treatment analysis of observed data (66 vs. 54 patients included), strategies did not significantly differ: −6 (SE 2) vs. −11 (SE 3), *p* = 0.13. For observed data, the estimated slopes of FIQ scores during the course of spa therapy indicated a tendency toward a difference between strategies: −5.2 points per week vs. −2.9 per week, *p* = 0.07. Estimated slopes during remote follow-up were both non-significant, without difference between strategies: −1.2 per year vs. + 2.4 per year, *p* = 0.36. In addition, a post hoc per protocol analysis (excluding all patients who discontinued strategy or follow-up) failed to demonstrate any significant difference between strategies. The rate of FIQ response (FIQ change < −14%) was 40% with SST vs. 47% with SST + TPE strategy (*p* = 0.40). Finally, the probability of FIQ response was increased in patients with higher systolic blood pressure (odds ratio (OR) 2.99, 95% confidence interval (CI_95%_) [1.41–6.35], *p* = 0.005) and a lower “magnification” dimension of Pain Catastrophizing score (0.80, [0.71–0.90], *p* = 0.0003) (Table S3).

### 3.4. Secondary Outcomes

We found no overall effect of either strategy on non-FIQ scores in the multivariate analysis of variance (*p* = 0.32). Between baseline and month 6, the proportion of patients not practicing any non-pharmacological treatment increased (26% to 35%), while the proportion of patients practicing treatment disrespecting EULAR recommendations decreased (65% to 58%), and the proportion of patients practicing treatment respecting EULAR recommendations remained unchanged (7% to 5%), without difference between strategies (*p* = 0.48) [73]. Meanwhile, the proportion of patients taking no drug at all increased (17% to 40%), as did the proportion of patients taking drugs neither recommended nor discouraged by EULAR (24% to 32%), and the proportion of patients taking discouraged drugs decreased (18% to 10%), as well as the proportion of patients taking recommended drugs (41% to 18%), without difference between strategies (*p* = 0.20) [73]. (See in Supplemental File for details, Tables S4 and S5, and for non-pharmacological treatments and drug categories, Figure S2).

### 3.5. Adverse Events

No death or pregnancy were reported during the trial. Nineteen serious adverse events were reported concerning 18 patients: 5 (6%) with SST and 13 (16%) with SST + TPE strategy, *p* = 0.08. Most events occurred during follow-up: 5 (100%) with SST and 12 (86%) with SST + TPE strategy, *p* = 1.00. No event was considered as related to SST strategy but three were related to SST + TPE strategy: one episode of acute low back pain leading to sciatica and surgery (certain causality) and two aggravations of depression (causality not excluded) (See Supplementary File, Table S5). Twenty-five patients encountered one painful episode during the trial and three patients encountered two. Among these 28 patients, 15 (19%) were with SST and 13 (16%) with SST + TPE strategy, *p* = 0.68. About two-thirds of the 31 episodes occurred during spa therapy in 20 patients (13%), without difference between strategies (10 vs. 10, *p* = 0.71). The probability of a first episode did not differ between strategies (Probability and CI_95%_ at month 6: 19% (11–29%) vs. 15% (9–26%), *p* = 0.56. (See in Supplemental File Figure S3.) Among the 249 symptoms of painful episodes reported, the number of symptoms per episode did not differ between strategies (median [interquartile range]: 6 (4–14) vs. 5 (2–11), *p* = 0.49), but there were more symptoms during spa therapy than during remote follow-up: 10 (4–16) vs. 3 (1–6), *p* = 0.03). Among the 20 episodes occurring during spa therapy, 14 (70%) led to modification of spa therapy treatments without difference between strategies (7 vs. 7, *p* = 1.00): spa treatments were modified for 2, spa therapy was discontinued temporarily for 9 and definitively for 3. Finally, 7 episodes (35%) led to an adaptation of drug treatment (4 vs. 3, *p* = 1.00) and none to adaptation of non-pharmacological treatment. We observed a higher risk of painful episodes when patients had a low diastolic blood pressure at baseline (per 10 mmHg less: OR = 4.7 and CI_95%_ 2.1–10.2], *p* = 0.0001), a higher FIQ (per 10 points more: 1.8 (1.2–2.7), *p* = 0.004), a normal body mass index (normal vs. thin or obese: 3.6 (1.3–10.1), *p* = 0.02) and a low income (below vs. above 1000€/month: 3.3 (1.2–9.1), *p* = 0.03).

### 3.6. Compliance to Strategies

Besides the 29 patients who completely stopped their participation, 89 patients (57%) had at least one prescribed spa treatment modified, without any significant difference between strategies (*p* = 0.87). Among the 11,304 spa treatments prescribed, 7% were cancelled and 1% modified. During the course of spa therapy, 69 (87%) patients with SST + TPE strategy declared having performed at least one indoor physical exercise session and 41 (52%) all three sessions. Concerning outdoor sessions: 60 declared (76%) attending at least one physical exercise session and 31 (39%) all three sessions. During remote follow-up, 76 (96%) patients completed at least one follow-up self-administered questionnaire on compliance with educational recommendations, and 64 (81%) completed all three questionnaires. At month 6, 87% declared practicing regular physical activity, 25% declared doing some physical exercise, 19% mental relaxation, and 23% taking pharmaceutical treatments. Ninety-four percent continued recommended physical exercises although 21% thought them painful, 10% useless, and 9% too constraining.

### 3.7. Efficacy of Spa Therapy

Pain intensity (−0.53%/week (SE 0.09)), fatigue (−1.36 (0.22)), sleepiness (−0.56 (0.14)), and impact of fibromyalgia (−3.77 (0.62)) significantly decreased during spa therapy (*p* ≤ 0.0001). All slopes were non-significantly different from zero during remote follow-up (all *p* between 0.40 and 0.88), suggesting a lasting benefit on fibromyalgia symptoms.

## 4. Discussion

This trial failed to demonstrate the superiority of the addition of a TPE program to a standard course of spa therapy for patients with fibromyalgia in terms of quality of life 6 months after the end of the spa therapy. This, despite having recruited slightly more patients than required by our sample size calculation and the close monitoring of follow-up resulting in fewer missing values for the primary outcome measure than anticipated and a post hoc analysis to try to identify subpopulations better responding to the intervention (−6 points on the FIQ while the expected result was −10 points). The result was confirmed by a worst-case sensitivity analysis. Considering the sample size and power, the observed lack of significance may be due a difference between strategies lower than expected. Furthermore, the small difference observed between strategies could be partially explained by contamination between strategies, although no more than 12 patients, among 4000 persons “taking the waters”, were treated in the same facility at the same time.

We did not undertake the educational assessments in both arms because these assessments are not only evaluation, but also motivation, so they constitute an intrinsic part of the intervention and we would have diluted the effects of the interventions.

Most of the patients were recruited through patient associations and/or their websites. This could have created a bias in favor of better informed, better treated, and better educated patients and, therefore, hyperselected. This highlights the importance of a broad recruitment of patients for such trials [50] and of personalization of the program(s) [57]. About half of our patients had already received EULAR-recommended drug treatment or had no medication. During the follow-up, the proportion of patients without any drugs increased, although the proportion using discouraged drugs remained unchanged. The absence of clearly identified disease mechanisms or efficient drug treatments could boost observed benefit from patient satisfaction at having received the add-on intervention, perhaps explaining the tendency toward a slight superiority of the SST + TPE strategy for most outcomes. This is a limitation of patient self-reported outcomes, particularly when there was no masking of the allocated strategy.

There were few deviations from the trial protocol. The effectiveness of a course of spa therapy on fibromyalgia symptoms at month 6, already reported by several authors, was confirmed for the patients in both strategies. It appears that the addition of TPE may not have been enough to significantly improve on the spa therapy alone, which provided a significant and lasting result and, therefore, was already too effective [75]. In contrast, the addition of spa therapy to a TPE program increases the efficiency of TPE [55,56,76,77]. The spa treatments were well tolerated with few adverse events. Only six patients, three per strategy, dropped out of the spa therapy course. Painful episodes were twice as frequent and their symptoms three times more frequent during spa therapy than during follow-up. However, symptoms were similar and spa-therapy-related fatigue should be considered. Though adverse events during spa therapy are seldom reported, the most prevalent, significant, and disabling ones are fatigue and unexplained pain exacerbation. We observed unsatisfactory tolerance and/or acceptance of SST + TPE strategy with 25% of patients dropping out. Three serious adverse events were deemed related to the SST + TPE strategy by the adjudication committee, but one was incidental and two occurred in patients with major prior histories of depression. The difference observed in the number of patients choosing to stop participation during spa therapy (one for SST vs. nine with SST + TPE) may be explained by the added fatigue from the TPE activities in the afternoon, although this difference also persisted during follow-up (0 vs. 14 for SST and SST + TPE respectively). The intervention stops at D18 in the SST arm while it continues until M12 in the SST + TPE arm. Consequently, it is impossible to stop the intervention after the cure in the SST arm, when it remains possible up to M12 in the SST + TPE arm. So, 20 patients (25%) with the SST + TPE strategy discontinued, suggesting either difficulties to continue when the dedicated support stopped or dissatisfaction with a program deemed insufficient. In addition, the high effectiveness of the spa treatment in itself made a slight effect of ETP less identifiable. We noticed that some patients with the SST + TPE strategy adopted individual changes in health behaviors, particularly the pursuit of physical activities back home, which suggests acceptability of TPE and its educative benefit. Physical activities (aerobic exercises, balneotherapy, multidisciplinary approach, and psychotherapy) are known to have relative effectiveness on quality of life, pain, fatigue, sleep, and depression [78]. The specific and precise training program, based on a multidisciplinary approach (indoor, outdoor, aquatic, and land physical activities), and practiced in a suitable, reassuring, and soothing environment, was well-tolerated and stimulating since at 6 months 87% of patients had continued physical activity. This confirms the interest of land-based exercise over water exercises to improve pain [79]. In addition, the program was above all innovative, combining physical activities on land and in the water, each providing benefits on the quality of sleep and the intensity of pain [80].

Self-administered questionnaires collected the patients’ perceptions of the intensity of their fibromyalgia symptoms. The different scores were strongly correlated making it difficult to weight the particular impact of each. Nevertheless, patients with a lower magnification on the Pain Catastrophizing Scale at baseline were more frequently responders, whatever the arm. The Pain Catastrophizing Scale assesses the patient’s state of mind regarding pain rather than the level of pain itself and depends more on a patient’s personality than on fibromyalgia severity. This could explain the significant association.

Patients with higher systolic blood pressure at baseline had a higher response rate (FIQ change from baseline < 14%) at month 6, and a higher risk of painful episodes was observed in patients with a low diastolic blood pressure, both emphasizing modifications in the reactions of the autonomic nervous system [81,82,83]. High blood pressure has been correlated with chronic pain [84,85] and patients with high blood pressure at baseline were those with more severe fibromyalgia. The risk of hypertension in this population could be related to a lower degree of baroreflex sensitivity [86], a good predictor of an increase in systolic blood pressure at 5 years [87]. Poverty [88,89] is known to be a risk factor of chronic pain and fibromyalgia, which could explain why it has been observed in the population of this study as a risk factor of painful episodes. In many countries, being overweight or obese are considered to be risk factors of chronic pain and fibromyalgia [65,90,91], although this was not the case in Korea [89]. These observations make it difficult to explain the increased risk of painful episodes in patients with a normal BMI, compared to the thin or obese. Further studies are needed on these possible predictors of painful episodes in the context of fibromyalgia.

This RCT showed different strengths. It was well-powered, with the number of enrolled patients meeting the target set in advance and based on a clinically relevant level of improvement. The follow-up procedures concerned both spa therapy and patient education. We intentionally used no generic quality-of-life questionnaire, such as SF36 or EQ-5D-L, because of the already large number (eight) of self-questionnaires. We had anticipated the problem of self-administered questionnaires being prone to missing data, in particular when completed remotely over the internet, and were able to keep missing d theta on FIQ changes below the expected rate using a small financial incentive.

Patients’ initial physical activity levels were not measured at baseline. The outcomes focused on clinical evolution and program acceptability rather than physical activity performance. Painful episodes and other adverse events were validated by an independent and experienced committee. Despite not being statistically conclusive regarding the main outcome, as a whole this trial offers a unique, complete, and robust set of data with a good level of evidence. While the main endpoint was evaluated at 6 months, the evaluation at 12 months showed that the modifications in lifestyle were maintained.

Nevertheless, the trial had some limitations. First, we overestimated the intrinsic effectiveness of patient education when defining the sample size. Furthermore, the patients’ existing pharmacological and non-pharmacological treatments already well-complied with EULAR recommendations, thus limiting the possibility of an added effect. The trial was open labelled and its outcomes of effectiveness were collected through self-administered questionnaires. These are inherently subjective and we wonder whether the small differences observed in favor of the SST + TPE strategy are the result of a true difference in effectiveness, or the self-deception of patients receiving this innovative strategy, as illustrated by the difference in evolution of pain intensity and pain relief: pain intensity remained constant over time but relief increased.

## 5. Conclusions

The FiETT trial showed that the addition of TPE to a course of spa therapy provided some extra benefit. Spa treatment has substantial and lasting beneficial therapeutic effects that therefore make it difficult to demonstrate a significant effect of a TPE program. Therapeutic education produced changes in individual health-promoting behaviors, which, according to recommendations, are essential for better coping with a chronic condition. After the intervention, the patients continued to practice different physical activities and increased their participations in everyday activities. A qualitative approach to the experience of the program would have been interesting.

We did not include a cost-effectiveness study in our trial because it was necessary to demonstrate the effectiveness of the intervention first. The results of the trial suggest that the trial be repeated at home with people just diagnosed and uninformed.

## Figures and Tables

**Table 1:** Baseline characteristics—FiETT trial, AFRETh.

Randomized Strategy				
			SST *N* = 78	SST + TPE *N* = 79
Gender	# (%)	Female	73 (94)	76 (96)
Age (years)	# (%)	[25–40] [40–60] [60–65]	5 (6) 60 (77) 13 (17)	6 (10) 54 (68) 19 (24)
Monthly net taxable income at baseline (€) *	# (%)	Unknown <1000 1000–1999 ≥2000	23 (29) 26 (33) 29 (37)	5 (6) 18 (23) 29 (37) 27 (34)
Socio-professional category	# (%)	Craftsperson, merchant, entrepreneur High-level executive, intellectual professional Employee Retired Other	11 (14) 10 (13) 38 (49) 6 (8) 13 (17)	2 (3) 11 (14) 40 (51) 8 (10) 18 (23)
Body mass index (kg/m^2^)	# (%)	[17–25] [25–30] [30–42]	38 (49) 24 (31) 16 (21)	42 (53) 18 (23) 19 (24)
Blood pressure	Mean (SE)	Systolic Diastolic	125 (1) 71 (1)	124 (1) 70 (1)
Patients with history medical event per MedDRA System Organ Class	# (%)	Musculoskeletal and connective Surgery tissue disorders Others	16 (21) 25 (32)	14 (18) 22 (28)
		Gastrointestinal disorders Appendectomy Others	10 (13) 16 (21)	12 (15) 11 (14)
		Vascular disorders	13 (17)	11 (14)
		Nervous system disorders	11 (14)	11 (14)
		Reproductive system and breast disorders	10 (13)	7 (9)
		Endocrine disorders Thyroid Diabetes	8 (10) 2 (3)	11 (14) 3 (4)
		Psychiatric disorders Depression Others	16 (15) 1 (1)	6 (8)
		Others	32 (41)	30 (38)
		None declared	20 (26)	27 (34)
Fibromyalgia Rapid Screening Tool	# (%)	5 6	14 (18) 64 (82)	10 (13) 69 (87)
Fibromyalgia Impact Questionnaire (%)	# (%)	Missing [0–45] [45–55] [55–65] [65–75] [75–100]	4 (5) 12 (15) 10 (13) 29 (37) 15 (4) 8 (2)	17 (22) 10 (13) 18 (23) 22 (6) 12 (3)
Fibromyalgia Impact Questionnaire	Mean (SE)		60 (13)	60 (16)
Pain intensity (Visual Analog Scale)	Mean (SE)		7 (0)	7 (0)
Kinesiophobia (Tampa Scale)	Mean (SE)		36 (1)	35 (1)
Pain Catastrophizing (Pain Catastrophizing Scale)	Mean (SE)		27 (1)	25 (2)
Rumination	Mean (SE)		9 (0)	8 (0)
Magnification	Mean (SE)		5 (0)	5 (0)
Helplessness	Mean (SE)		14 (1)	12 (1)
Functional limitation due to arthritis (WOMAC scale)	Mean (SE)		36 (1)	36 (1)
Fatigue (Pichot scale)	Mean (SE)		23 (1)	24 (1)
Sleepiness (Epworth scale)	Mean (SE)		12 (1)	12 (1)
Anxiety (Hospital Anxiety and Depression scale)	Mean (SE)		12 (1)	13 (0)
Depression (Hospital Anxiety and Depression scale)	Mean (SE)		10 (1)	8 (1)
Patients with non-pharmaceutical treatment for fibromyalgia compliant with EULAR 2016	# (%)	None declared Recommended only At least one discouraged	16 (21) 5 (6) 57 (73)	25 (32) 7 (9) 47 (59)
Patients with drugs for fibromyalgia compliant with EULAR 2016	# (%)	None declared Recommended only At least one discouraged	13 (17) 51 (65) 14 (18)	14 (18) 51 (65) 14 (18)

SST: standardized spa therapy; TPE: therapeutic patient education; *N*: number of patients; #: number of patients with a certain characteristic; SE: standard error; WOMAC: Western Ontario and McMaster Universities Arthritis index; MedDRA: Medical Dictionary for Regulatory Activities; EULAR: European Alliance of Associations for Rheumatology. * Net monthly taxable income was around 1140 € in France in 2015.

## Data Availability

The data presented in this study are available on request from the corresponding author. The data are not publicly available due to privacy.

## Supplementary Materials

The following are available online at https://www.mdpi.com/article/10.3390/ijerph19084613/s1, Table S1: Simultaneous course of spa therapy and therapeutic patient education program—FiETT trial, AFRETh, Table S2: Mean change from baseline in Fibromyalgia Impact Questionnaire—FiETT trial, AFRETh, Table S3: Determinants of response (FIQ change from baseline to month 6 < −14%)—FiETT trial, AFRETh, Table S4: Evolution of secondary outcomes—FiETT trial, AFRETh, Table S5: Supplemental results on secondary outcomes—FiETT trial, AFRETh, Figure S1: Patients’ flowchart—FiETT trial, AFRETh, Figure S2: Proportion of patients in SST vs. SST + TPE strategy changing treatment category from baseline to month 6 according to EULAR (European Alliance of Associations for Rheumatology) 2016 recommendations—FiETT trial, AFRETh, Figure S3: Probability of painful episodes from baseline to month 12 (Kaplan-Meier)—FiETT trial, AFRETh.

## Abreviations

AFRETh	Association Française de Recherche sur le Thermalisme (French society for thermal spa research)
BDI	Beck Depression Inventory
CIS	Checklist Individual Strength
eCRF	electronic Case Report Form
EULAR	European Alliance of Associations for Rheumatology
FiETT	Fibromyalgie et Education Thérapeutique en cure Thermale (Fibromyalgia and Therapeutic Education during thermal spa cure)
FIQ	Fibromyalgia Impact Questionnaire
FIRST	Fibromyalgia Rapid Screening Test
GTPS	Graded Tender Point Score
HAD	Hospital Anxiety and Depression scale
MedDRA	Medical Dictionary for Regulatory Activities
mmHg	millimeter of mercury
MPQ	McGill Pain Questionnaire
PCS	Pain Catastrophizing Scale
PSQI	Pittsburgh Sleep Quality Index
SE	Standard Error
SF-36	Short Form 36
SOC	System Organ Class
SST	Standardized Spa Therapy
TSK	Tampa Scale of Kinesiophobia
TPE	Therapeutic Patient Education
VAS	Visual Analog Scale
WOMAC	Western Ontario and McMaster scale
