# Supplementary material for: Therapeutic Patient Education for Fibromyalgia during Spa Therapy: The FiETT Randomized Controlled Trial
Source: Int J Environ Res Public Health. 2022 Apr 11;19(8):4613. doi: 10.3390/ijerph19084613 (PMC9030628; doi:10.3390/ijerph19084613)
Supplement: Supplementary file 1 [file ijerph-19-04613-s001.zip › ijerph-1601282-supplementary.pdf]

Table S1. Simultaneous course of spa therapy and therapeutic patient education program - FiETT trial, AFRETh.

Main objective

To live better with fibromyalgia by becoming a competent partner knowing how to increase and adapt activities.
During the program, the patient should acquire skills about fibromyalgia, understand the value of regular physical exercise and put it into practice and plan over time.

Context

the patients come to Dax, are accommodated in thermal establishments and / or hotels or in housing in town. Treatments are all carried out daily for 18 days (legal duration validated by French public health authorities). The control group receives Spa cares. The intervention group receives the same Spa cares and in addition the Therapeutic Patient Education.

**Outlines**	**Spa therapy**<br>*for SST and SST+TPE arms*	**Therapeutic Patient Education**<br>*for SST+TPE arm*
**Organization**	**Overall**<br>*. Duration 3 weeks = 18 consecutive days except Sunday (as usual in France)*<br>*. Reimbursed by the French health insurance*<br>*. Patients' accommodation in spa establishments of Dax resort providing both thermal cares and hotel services*<br>**Spa cares**<br>*. Prescribed by spa physician the day before spa therapy started*<br>*. Dispensed in the mornings and spread out over the 18 days by spa establishments staff*<br>**Oversight**<br>*. Follow-up and adaptation by same spa nurse (1 / spa establishment)*<br>*. Supervision by same spa physician during 3 thermal assessments*	**Overall**<br>*. Duration 3 weeks = 18 consecutive days except Sunday (as usual in France)*<br>*. TPE standardized program (Fibr'Eaux), developed by spa physicians of Dax resort and approved by French public health agencies*<br>*. Payed by FiETT trial, during trial follow-up for patients in SST+TPE arm, or the following year for patients in SST arm coming back for their next spa therapy*<br>**Activities**<br>*. Prescribed by spa physician the day before spa therapy started*<br>*. Dispensed in the afternoons and spread out over the 18 days by 2 TPE nurses, 4 facilitators and 2 physical coaches trained in TPE*<br>**Oversight**<br>*. Follow-up by TPE nurse*<br>*. Supervision by same spa physician during 3 educational assessments*
**Before spa therapy**	**First thermal assessment** / spa physician<br>*. Clinical exam and interview*<br>*. Validation of fibromyalgia diagnosis*<br>*. Checking of absence of contraindication to heat treatments*<br>*. Prescription of personalized program*	**First educational assessment** / spa physician<br>*. Situation, abilities, participation in daily life*<br>*. Desires and motivation*<br>*. Limitations to physical exercise co-assessed by physical coach*<br>→ Personalized program
**During spa therapy**<br>***3 weeks***	**72 spa treatments** / thermal care facilities staff<br>*. Individual bathing in mineral water with underwater shower (36°C, 10-20 min)*<br>*. Mineral mud application (40-42°C, up to 15 min)*<br>*. Massages by registered physiotherapist (36°C, up to 10 min)*<br>*. Collective rehabilitation in mineral pool supervised by registered physiotherapist (32-34°C, 20 min)*	**6 educational workshops** / always the same facilitators trained in TPE<br>*. 4 mandatory Understanding the disease, Moving well, Managing pain and stress, Sleeping well*<br>*. 2 optional Using medications well, Living with the disease*<br>*. In the afternoons, 2 workshops per week*<br>*. Collective, 1.5 to 2 hours each*

	. In the mornings, 24 treatments per week<br>*. With adjustment to patient's limitations during the cares*<br><br>**Intermediate thermal assessment** / spa physician<br>*. Clinical exam and interview*<br>*. Review of additional adjustments on the spot*<br>*. Adjustment of personalized program*	**6 physical exercise sessions** / always the same trained physical coaches<br>*. 3 indoor Fibrotherapy®* [1]<br>*. 3 outdoor aerobic activities* — *Walking 500 to 1,000 m*<br>*8 rehabilitation exercise stations*<br>*3 rest stations*<br>*. In the afternoons, 1 indoor + 1 outdoor sessions per week*<br>*. Collective, 1 hour each*<br>*. With adjustment to patient's limitations*
**At end of spa therapy**	**Final thermal assessment** / spa physician<br>*. Clinical exam and interview*<br>*. Review of additional adjustments on the spot*<br>*. Reporting to corresponding physicians*	**Second educational assessment** / spa physician<br>*. Clinical exam and interview*<br>*. Review of knowledge acquisition and physical progress*<br>*. Advice on proper reaction to painful episode*<br>*. Elaboration of an action plan to take back home*<br>*. Reporting to corresponding physicians*
**3 month after spa therapy**		**Third educational assessment** / spa physician<br>*. Mailing of educational workshops material and phone interview*<br>*. Review of action plan implementation*<br>*. Assessment of physical activity maintenance and quality of life*<br>*. Suggestions for improvement*

[1] *Fibrotherapy® is a registered trade mark for a set of stretching and relaxing exercises adapted to fibromyalgia and led by a trained and certified coach.*

Table S2. Mean change from baseline in Fibromyalgia Impact Questionnaire - FiETT trial, AFRETh.

**DATASET**	**OBSERVED DATA**							**MISSING = FAILURE** principal analysis					**WORST CASE FAVORING SST**					**WORST CASE FAVORING SST+TPE**				
**STRATEGY**	**SST** N = 78			**SST+TPE** N = 79				**SST** N = 78		**SST+TPE** N = 79			**SST** N = 78		**SST+TPE** N = 79			**SST** N = 78		**SST+TPE** N = 79		
	**M**	**Mean**	**SE**	**M**	**Mean**	**SE**	**p-value**	**Mean**	**SE**	**Mean**	**SE**	**p-value**	**Mean**	**SE**	**Mean**	**SE**	**p-value**	**Mean**	**SE**	**Mean**	**SE**	**p-value**
**FOLLOW-UP**																						
day 0	4	0	(0)	0	0	(0)		0	(0	0	(0)		0	(0)	0	(0)		0	(0)	0	(0)	
day 18	12	-10	(2)	9	-14	(2)		) -7	(2)	-11	(2)		-13	(2)	-11	(2)		-7	(2)	-17	(2)	
month 3	11	-5	(2)	8	-12	(2)		-2	(2)	-10	(2)		-8	(2)	-10	(2)		-2	(2)	-15	(2)	
month 6	10	-6	(2)	5	-10	(2)	0.17	-3	(2)	-9	(2)	0.06	-8	(2)	-9	(2)	0.96	-3	(2)	-11	(2)	0.005
month 12	14	-6	(2)	7	-15	(3)		-3	(2)	-13	(3)		-10	(2)	-13	(3)		-3	(2)	-18	(3)	

SST: Standardized Spa Therapy; TPE: Therapeutic Patient Education; N: number of patients; M: number of missing values; SE: Standard Error

Table S3. Determinants of response (FIQ change from baseline to month 6 < -14%) - FiETT trial, AFRETh.

			Univariate Analysis[1]			Multivariable Analysis[2]		
			OR	$CI_{95\%}$	p-value	OR	$CI_{95\%}$	p-value
**Strategy**		SST+TPE vs SST	1.36	0.70-2.55	0.36			
**Gender**		Female vs Male	0.44	0.10-1.94	0.28			
**Age** – *Years*		[25-40[vs [60-65[	0.93	0.24-3.59	0.46			
		[40-50[	0.44	0.16-1.27				
		[50-60[	0.74	0.31-1.77				
**Body Mass Index** – *Kg/m²*		[17-25[vs [25-42]	0.81	0.42-1.57	0.53			
**FIRST**		5 vs 6	1.51	0.60-3.82	0.38			
**Net Average Monthly Income** – €		< 1,000	0.91	0.39-2.14	0.93			
		1,000-2,000	0.86	0.39-1.90				
		≥ 2,000	1.00	-				
**Professional Status**		Active	0.65	0.30-1.41	0.62			
		Inactive	0.88	0.35-2.21				
		Other	1.84	0.29-7.13				
		Incapacitated	1.00	-				
**Socio-Professional Category**		Executive, intellectual profession	0.82	0.26-2.59	0.88			
		Farm operator, craftsman, shopkeeper, business owner, intermediate profession	0.62	0.18-2.08				
		Employee, worker	0.91	0.40-2.10				
		Retired, inactive, other	1.00	-				
**Blood Pressure** - *cm Hg*	**Systolic**	[13-16] vs [10-12]	**2.50**	**1.26-4.98**	**9.10^{-3}**	**2.99**	**1.41-6.35**	**5.10^{-3}**
	**Diastolic**	[8-9] vs [6-7]	0.78	0.38-1.58	0.48			
**Joints limited range of motion**								
Cervical vertebrae		With vs Without	1.29	0.64-2.61	0.48			
Dorsal vertebrae		With vs Without	1.32	0.68-2.57	0.41			
Lumbar vertebrae		With vs Without	1.09	0.56-2.15	0.79			
Shoulders		With vs Without	1.10	0.57-2.14	0.78			
Elbows		With vs Without	1.15	0.50-2.64	0.74			
Wrists		With vs Without	**1.64**	**0.79-3.43**	**0.19**	2.13	0.92-4.95	*0.08*

Hands	With vs Without	0.90	0.42-1.93	0.78			
Hips	With vs Without	0.99	0.51-1.97	0.99			
Kneels	With vs Without	**1.67**	**0.83-3.38**	**0.15**			*0.68*
Ankles	With vs Without	1.13	0.53-2.41	0.74			
**Pain Intensity** – Visual Analog Scale on 10 points		1.06	0.89-1.26	0.53			
**Kinesiophobia** – Tampa Kinesiophobia Scale on 64 points		**0.96**	**0.92-0.99**	**0.04**			*0.94*
**Pain Catastrophizing** – Pain Catastrophizing Scale on 52 points							
Rumination on 16 points		**0.88**	**0.80-0.96**	**4.10^{-3}**			*0.86*
Magnification on 12 points		**0.81**	**0.73-0.91**	**4.10^{-4}**	**0.80**	**0.71-0.90**	**3.10^{-4}**
Helplessness on 24 points		**0.91**	**0.86-0.96**	**8.10^{-4}**			*0.47*
**Functional Limitations** – WOMAC physical function on 65 points		**0.98**	**0.95-1.01**	**0.14**			*0.16*
**Fatigue** – Pichot Fatigue scale on 32 points		**0.92**	**0.86-0.98**	**8.10^{-3}**			*0.14*
**Sleepiness** - Epworth Sleepiness scale on 24 points		0.98	0.82-1.03	0.39			
**Anxiety** – Hospital Anxiety and Depression scale on 21 points		**0.92**	**0.85-0.99**	**0.03**			*0.91*
**Depression** – Hospital Anxiety and Depression scale on 20 points		**0.92**	**0.85-0.99**	**0.03**			*0.81*

OR: Odds Ratio; $CI_{95\%}$: 95% Confidence Interval; SST: Standardized Spa Therapy; TPE: Therapeutic Patient Education Patient; FIRST: Fibromyalgia Rapid Screening Test; WOMAC: West Ontario and McMaster Universities Arthritis Index

[1] *Significant level 0.25; Bold: estimates and p-values of variables retained for multivariable analysis*

[2] *Backward selection, significant level 5%; Italic: estimates and p-values at removal from the model; Bold: estimates and p-values in the final model*

Table S4. Evolution of secondary outcomes - FiETT trial, AFREThr.

		STRATEGY								
		SCT				SCT+TPE				*p at M06*
		**D00**	**D18**	**M06**	**M12**	**D00**	**D18**	**M06**	**M12**	
**Non-FIQ scores (%)**	*#; Mean (SE)*									
Pain (VAS)										
Intensity		70; 7 (0)	68; 5 (0)	70; 6 (0)	65; 6 (0)	77; 7 (0)	72; 5 (0)	74; 6 (0)	71; 5 (0)	*0.58*
Relief		-	60; 4 (0)	70; 3 (0)	65; 3 (0)	-	64; 5 (0)	74; 4 (0)	71; 5 (0)	*0.03*
Kinesiophobia (TKS)		73; 36 (1)	69; 35 (1)	70; 37 (1)	65; 38 (1)	79; 35 (1)	73; 31 (1)	74; 34 (1)	72; 33 (1)	*0.17*
Pain catastrophizing (PCS)		72; 27 (1)	69; 24 (2)	69; 24 (1)	65; 24 (2)	79; 25 (2)	72; 18 (1)	73; 20 (2)	72; 17 (2)	*0.74*
Rumination		72; 9 (0)	69; 8 (1)	69; 8 (0)	65; 8 (1)	79; 8 (0)	72; 7 (0)	73; 7 (0)	72; 6 (0)	*0.99*
Magnification		72; 5 (0)	69; 4 (0)	68; 4 (0)	65; 4 (0)	79; 5 (0)	72; 3 (0)	73; 3 (0)	70; 3 (0)	*0.71*
Helplessness		72; 14 (1)	68; 12 (1)	68; 12 (1)	65; 12 (1)	79; 12 (1)	72; 8 (1)	73; 10 (1)	70; 8 (1)	*0.32*
Functional limitations (WOMAC)		72; 36 (1)	69; 30 (2)	69; 33 (1)	65; 32 (1)	79; 36 (1)	72; 29 (1)	73; 32 (1)	72; 29 (2)	*0.63*
Fatigue (Pichot)		71; 23 (1)	69; 21 (1)	69; 22 (1)	65; 22 (1)	79; 24 (1)	72; 17 (1)	73; 20 (1)	72; 18 (1)	*0.001*
Sleepiness (Epworth)		72; 12 (1)	69; 11 (1)	69; 10 (1)	65; 10 (1)	79; 12 (1)	72; 10 (1)	73; 10 (1)	72; 10 (1)	*0.51*
Anxiety and depression (HAD)										
Anxiety		73; 12 (1)	69; 11 (1)	69; 10 (1)	65; 10 (1)	79; 13 (0)	73; 9 (1)	73; 10 (1)	71; 9 (1)	*0.14*
Depression		73; 10 (1)	69; 9 (1)	69; 10 (1)	65; 9 (1)	79; 8 (1)	73; 6 (1)	72; 7 (1)	71; 7 (1)	*0.27*
**Blood pressure**										
Blood pressure (mmHg)	*N*	78	76			79	76			*NA*
Systolic	*Mean (SE)*	125 (1)	123 (1)			124 (1)	121 (1)			
Diastolic		71 (1)	70 (1)			70 (1)	69 (1)			
Hypertension severity - ESC/ESH 2018	*N*	78	76			79	77			*NA*
Optimal	*# (%)*	7 (9)	14 (18)			17 (22)	18 (24)			
Normal		47 (60)	48 (63)			40 (51)	45 (60)			
Normal high		15 (19)	6 (8)			12 (15)	9 (12)			
Mild hypertension		2 (3)				1 (1)	1 (1)			
Isolated systolic hypertension		7 (9)	8 (11)			9 (11)	3 (4)			
**Joints troubles**										
Joints with limited range of motion	*N*	78	76			79	77			*NA*
Cervical vertebra	*# (%)*	48 (62)	26 (34)			54 (68)	31 (40)			
Dorsal vertebra		39 (50)	14 (18)			37 (47)	17 (22)			
Lumbar vertebra		46 (59)	20 (26)			45 (57)	23 (30)			
Shoulders		40 (51)	17 (22)			44 (56)	24 (31)			

		STRATEGY								
		SCT				SCT+TPE				*p at*
		D00	D18	M06	M12	D00	D18	M06	M12	*M06*
Elbows		16 (21)	9 (12)			15 (19)	6 (8)			
Wrists		19 (24)	6 (8)			24 (30)	9 (12)			
Hands		17 (22)	6 (8)			23 (29)	7 (9)			
Hips		29 (37)	7 (9)			32 (41)	14 (18)			
Knees		25 (32)	8 (11)			27 (34)	11 (14)			
Ankles		18 (23)	7 (9)			20 (25)	8 (10)			
Joint stiffness and muscle contractures										
(Number of locations)	*N*	78	76			79	77			*NA*
0	*# (%)*	15 (19)	37 (49)			7 (9)	22 (29)			
1		20 (26)	14 (18)			21 (27)	27 (35)			
2 to 4		31 (40)	22 (19)			32 (41)	21 (27)			
5 or more		12 (15)	6 (8)			19 (24)	7 (9)			
**Non-pharmacological cares**										
Nature	*N*	78		76	76	79		66	65	
None	*# (%)*	16 (21)		23 (30)	28 (37)	25 (32)		26 (39)	24 (37)	*0.30*
Physiotherapy		51 (65)		43 (57)	36 (47)	45 (57)		36 (55)	32 (49)	*0.87*
Osteopathy		10 (13)		5 (7)	4 (5)	3 (4)		4 (6)	3 (5)	*1.00*
Acupuncture		15 (19)		12 (16)	10 (13)	11 (14)		10 (15)	9 (14)	*1.00*
Sophrology		12 (15)		8 (11)	8 (11)	7 (9)		6 (9)	6 (0)	*1.00*
Hypnosis		10 (13)		10 (13)	6 (8)	8 (10)		3 (5)	5 (8)	*0.09*
Others		30 (38)		29 (38)	20 (26)	20 (25)		21 (32)	24 (37)	*0.49*
Compliance with EULAR recommendations	*N*			76	76	79		66	65	*0.49*
At least one non-compliant	*# (%)*	55 (71)		45 (59)	38 (50)	47 (59)		37 (56)	35 (54)	
None taken		16 (21)		23 (30)	28 (37)	25 (32)		26 (39)	24 (37)	
All compliant		4 (5)		5 (7)	8 (11)	7 (9)		2 (3)	4 (6)	
None assessed		3 (4)		3 (4)	2 (3)	0 (0)		1 (2)	2 (3)	
**Drugs**										
Class	*N*	78		76	76	79		68	63	
None taken	*# (%)*	13(17)		26 (34)	39 (51)	14 (18)		32 (47)	33 (52)	*0.13*
Non-steroidal anti-inflammatory drugs		7 (9)		5 (7)	5 (7)	7 (9)		6 (9)	4 (6)	*0.76*
Analgesics level 1		30 (38)		27 (36)	16 (21)	29 (37)		17 (25)	12 (19)	*0.21*
Analgesics level 2		26 (33)		16 (21)	18 (24)	30 (38)		13 (19)	15 (24)	*0.84*
Analgesics level 3		4 (5)		1 (1)	2 (3)	3 (4)		2 (3)	3 (5)	*0.61*

		STRATEGY								
		SCT				SCT+TPE				***p at***
		**D00**	**D18**	**M06**	**M12**	**D00**	**D18**	**M06**	**M12**	***M06***
Antidepressants		40 (51)		25 (33)	23 (30)	39 (49)		14 (21)	17 (27)	*0.14*
Anti-epileptics		13 (17)		9 (12)	7 (9)	13 (16)		5 (7)	6 (10)	*0.42*
Corticoids						1 (1)				
Hypnotics		10 (13)		4 (5)	6 (2)	10 (13)		5 (7)	4 (6)	*0.74*
Anxiolytics		16 (21)		9 (12)	6 (8)	11 (14)		5 (7)	6 (10)	*0.42*
Muscle relaxers		4 (5°		2 (3)	2 (3)	4 (5)		1 (1)	1 (2)	*1.00*
Others		11 (14)		22 (29)	18 (24)	12 (15)		12 (18)	11 (17)	*0.13*
Compliance with EULAR recommendations	*N*	78		76	76	79		68	63	*0.47*
At least one non-compliant	*# (%)*	14 (18)		8 (11)	8 (11)	14 (18)		6 (9)	5 (8)	
None taken		13 (17)		26 (34)	39 (51)	14 (18)		32 (47)	33 (52)	
All compliant		31 (40)		16 (21)	15 (20)	34 (43)		10 (15)	13 (21)	
None assessed		20 (26)		26 (34)	14 (18)	17 (22)		20 (29)	12 (19)	

VAS: Visual Analogue Scale; TKS: Tampa Kinesiophobia Scale; PCS: Pain Catastrophizing Scale; WOMAC: West Ontario and McMaster Universities Arthritis Index scale; HAD: Hospital Anxiety and Depression scale; ESC: European Society of Cardiology; ESH: European Society of Hypertension; EULAR: European Alliance of Associations for Rheumatology; SE: Standard Error; N: Number of patients; #: Number of patients with characteristic; %: percentage of patients with characteristic; NA: Not Applicable.

Table S5. Supplemental results on secondary outcomes - FiETT trial, AFRETh.

Outcome	Results
Satisfaction about spa therapy	Among the 90% patients having expressed their opinion, satisfaction on the spa therapy session was 89% (knowledge of spa centers staff dispensing cares on fibromyalgia 86% vs 92%, p=0.30), and 90% to 100% on all other items, without difference between strategies (p 0.37 to 1.00).
Modification of non-pharmaceutical treatments	Modified non-pharmaceutical treatments were mainly mud-packs (24%), individual bath with immersed and pressure varying hydromassage (22%), individual aerobath (24%) and mobilization in the pool (15%), p-value=0.28. Duration was reduced in 79% spa treatments (70% vs 88%) and extended in 10% (18% and 2%), p-value<10^{-4}. Location was reduced in 18% (11% vs 25%), p-value=0.002. Suppressed treatments were not replaced in 87% (82% vs 91%) or replaced mainly by individual bath with immersed and pressure varying hydromassage in 5% (10% vs 1%), p-value<10^{-4}).
Maintain of equipoise	Despite our recommendations to maintain equipoise when interacting with the patient, 48% of spa physicians (41% vs 56%, p-value=0.21), 55% of sites nurses (47% vs 63%, p-value=0.06), 22% of physiotherapists (15% vs 29%, p-value=0.08), and 61% of Fibr'Eaux nurses (SST+TPE strategy) expressed an opinion on the Fibr'Eaux program, and always in favor of its efficacy.
Physical exercise in SST+TPE strategy	During spa therapy session, the proportion of patients having done the indoor exercises raised along time: 46% to 87% depending on exercise on first session, 68% to 95% on second session and 93% to 98% on third session. The proportion of patients having done the outdoor exercises increased and decreased: 82% to 91% depending on exercise on first session, 85% to 100% on second session and 78% to 100% on third session.

Modification of lifestyle habits	During remote follow-up, 58% to 70% patients declared managing stress, and pain through managing stress; 60% to 86% declared taking precautions to favor sleepiness in the evening (regular schedule, no excitement, soothing environment), but only 43% to 46% during the day (invigorating awakening, long nap); 51% could discuss fibromyalgia with their family, 70% with their therapists, but only 37% with their occupational physician; 41% with the Department House for Disabled People; 15% appealed for Social Services and 29% are member of a patients' association
Global opinion on TPE	Among patients in SST+TPE strategy, 87% found TPE was efficacious, 15% reckoned they reached their initial objectives fully, and 68% partially.
Symptoms of painful episodes	Forty-two of the 51 symptoms proposed in the eCRF were selected at least once, and the most frequent were: muscle pains (9%), joint pains (8%), fatigue and exhaustion (6% each), cephalgia (5%), muscle weakness, bout of depression, sleep disorders and joint stiffness (4% each), nausea, muscle stiffness, anxiety and memory and attention disorders (3% each). Strategies did not differ on symptoms characteristics: 57% had forerunners (p-value=0.79); 15% were new, 23% were a relapse and 58% an aggravation of a former or present symptom (p-value=0.33).
Nature of the 3 serious adverse reactions	After an afternoon uncomfortably seating on a chair too narrow during an education workshop, a patient with a 41.5 Kg/m^2 body mass index developed acute lumbago, later progressing to sciatica and surgery for herniated disk 1 week before month 6 (certain causality).<br>One patient was hospitalized for depression and one had an aggravation of depression, both 6 weeks after end of spa therapy (causality not excluded).

SST: Standardized Spa Therapy; TPE: Therapeutic Patient Education

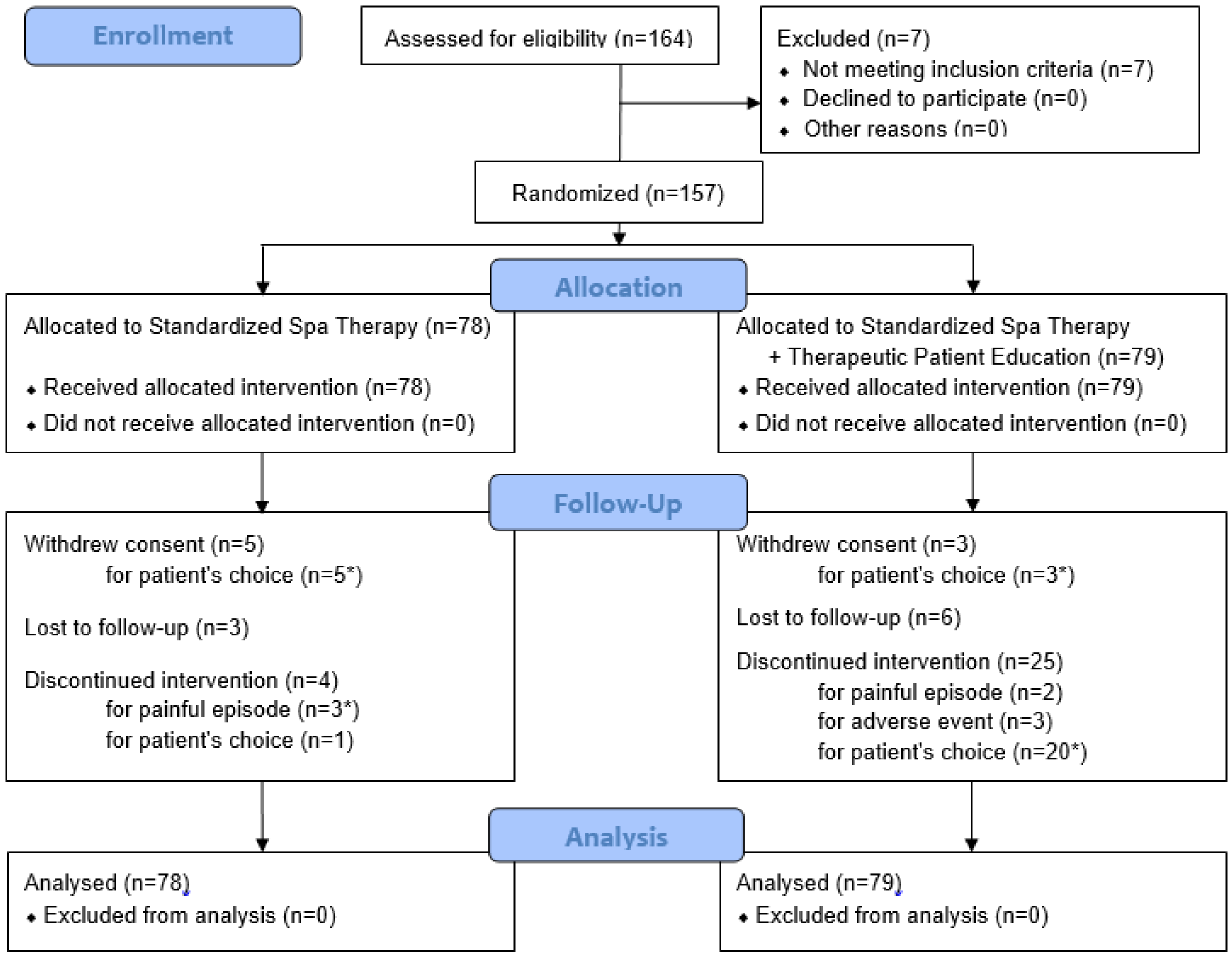

**One patient in each strategy first discontinued strategy then withdrew consent.*

Figure S1. Patients' flowchart - FiETT trial, AFRETh

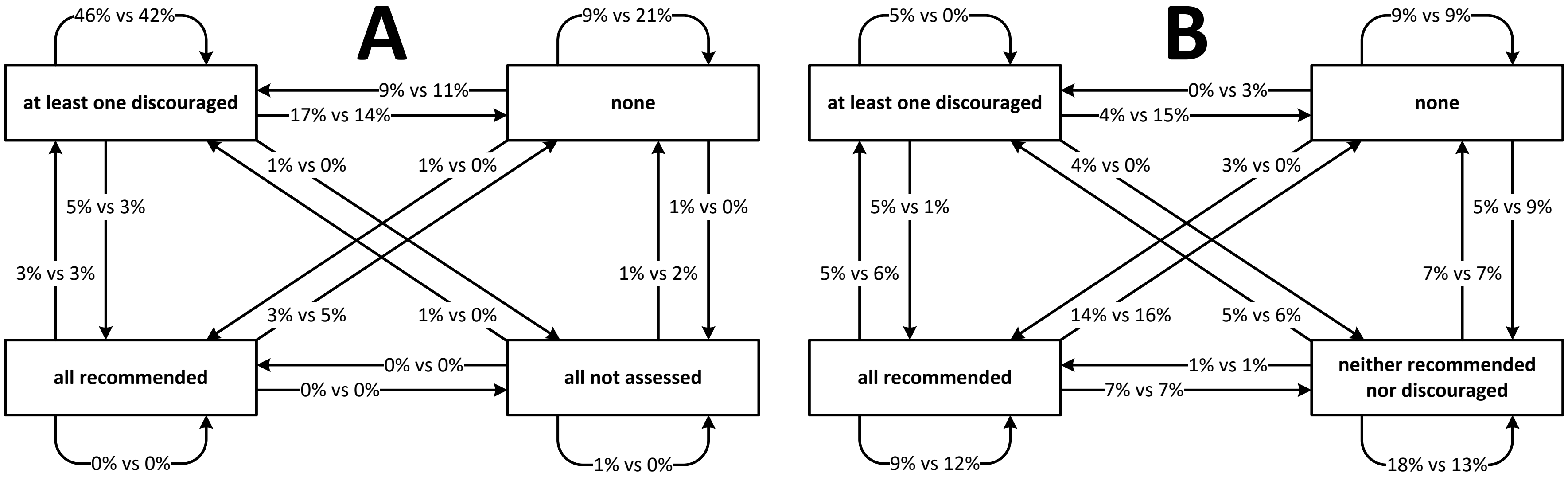

Figure S2. Proportion of patients in SST vs SST+TPE strategy changing treatment category from baseline to month 6 according to EULAR (European Alliance of Associations for Rheumatology) 2016 recommendations - FiETT trial, AFRETh.

SST: Standardized Spa Therapy; TPE: Therapeutic Patient Education; Panel A: Non-pharmaceutical treatments; Panel B: Pharmaceutical treatments

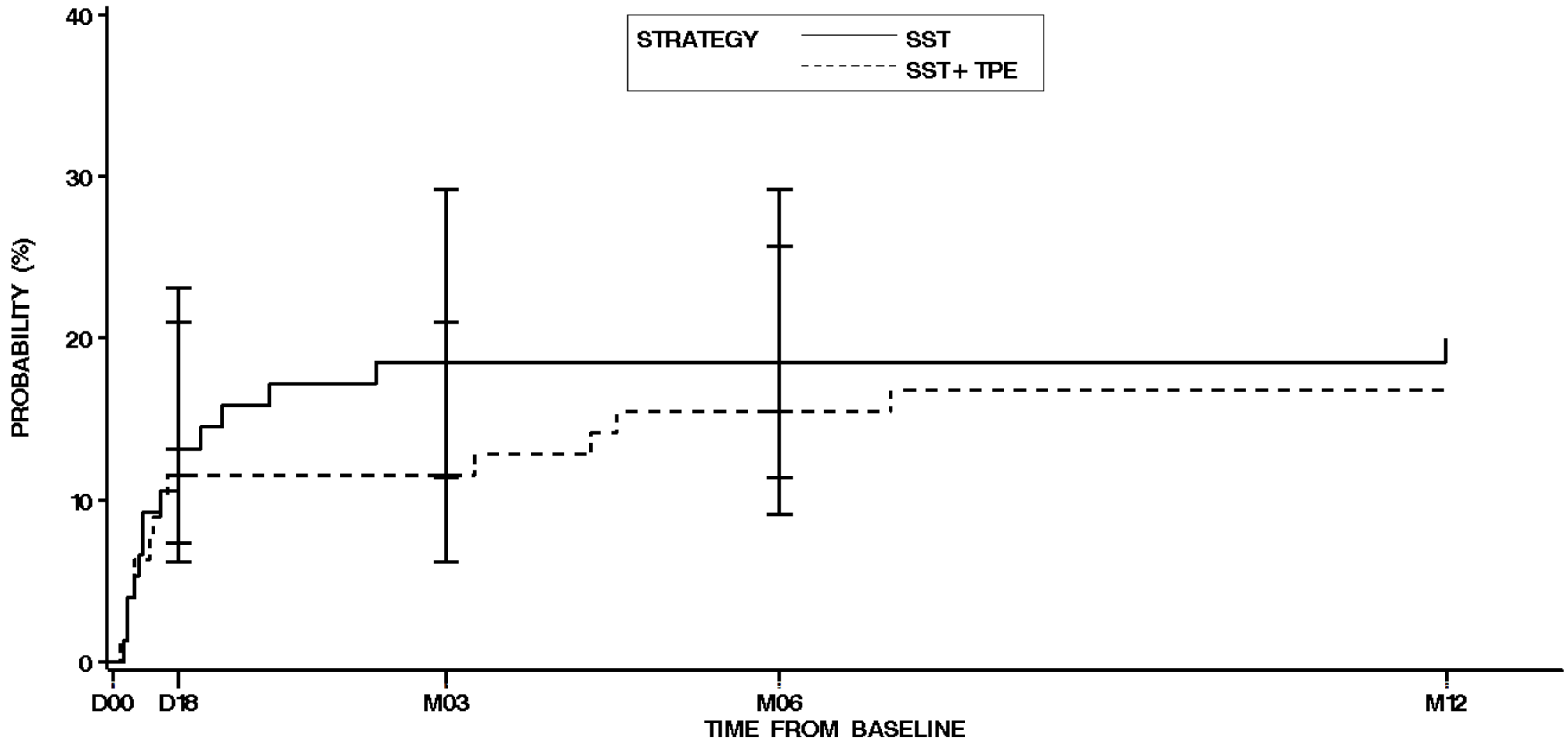

Figure S3. Probability of painful episodes from baseline to month 12 (Kaplan-Meier) - FiETT trial, AFRETh.

SST: Standardized Spa Therapy; TPE: Therapeutic Patient Education
